# Feasibility of combining tDCS with exercise for increasing physical activity in people with depression

**DOI:** 10.3389/fphys.2025.1597234

**Published:** 2025-08-25

**Authors:** Ana M. Abrantes, Julia Browne, Mascha van ‘t Wout-Frank, Linda L. Carpenter, Michael D. Stein, Lisa A. Uebelacker, Daniel Audet, Julie A. Desaulniers, Sarah Garnaat

**Affiliations:** ^1^ Butler Hospital, Providence, RI, United States; ^2^ Department of Psychiatry and Human Behavior, Alpert Medical School of Brown University, Providence, RI, United States; ^3^ Center of Innovation on Transformative Health Systems Research to Improve Veteran Equityand Independence (THRIVE COIN), VA Providence Healthcare System, Providence, RI, United States; ^4^ Department of Health Law, Policy and Management, Boston University School of Public Health, Boston, MA, United States; ^5^ Department of Psychiatry, Dartmouth-Hitchcock Medical Center and Geisel School of Medicine, Lebanon, NH, United States

**Keywords:** transcranial direct current stimulation, aerobic exercise, physical activity, depression, feasibility

## Abstract

**Introduction:**

Physical inactivity and depression are significant public health concerns, often co-occurring and exacerbating one another. Transcranial direct current stimulation (tDCS) has shown promise in enhancing cognitive and affective processes, potentially improving exercise adherence and outcomes in individuals with depressive symptoms. This study aimed to evaluate the feasibility and preliminary within group effects of combining tDCS with an aerobic exercise (AE) intervention to increase physical activity in individuals with elevated depressive symptoms.

**Method:**

A pilot randomized controlled trial (RCT) was conducted with 51 participants exhibiting low physical activity levels and elevated depressive symptoms. Participants were randomized to receive either active tDCS (n = 25) targeting the left dorsolateral prefrontal cortex (DLPFC) or sham tDCS (n = 26), followed by supervised AE sessions three times per week for eight weeks. Physical activity was measured using accelerometers, and secondary outcomes included cardiorespiratory fitness, depressive symptoms, and affect.

**Results:**

Feasibility metrics indicated moderate adherence rates to sessions, good follow-up rates, and successful blinding as belief about receiving active stimulation was comparable across conditions. Within the active tDCS group, small-to-medium effect sizes were observed for increases in accelerometer-derived daily steps (d = 0.36) and MVPA (d = 0.34) at end of treatment. Both the active tDCS and sham groups demonstrated large within-group improvements in cardiorespiratory fitness (d = 0.99 for active, d = 1.18 for sham) and self-reported MVPA (active d = 0.78, sham d = 0.90). Similarly, large reductions in depressive symptoms (active d = −1.00, sham d = −0.88) were observed within both groups.

**Discussion:**

The combination of tDCS and AE appears feasible and shows preliminary potential for positively influencing daily step counts in individuals with depressive symptoms. The results support further investigation into tDCS as an adjunctive treatment to enhance exercise outcomes in this population.

## 1 Introduction

Physical inactivity remains a paramount public health issue, with only half of the United States population meeting recommended activity levels (Martinez-Gomez et al., 2024). Regular exercise is not only associated with reduced risks of cancers including breast, endometrial, prostate, and bladder but also protects against cardiovascular diseases, type 2 diabetes, metabolic syndrome, bone loss, and overweight issues (Lee et al., 2012; Cunningham et al., 2020). Further, exercise, particularly at appropriate intensities, is known to positively impact mental health (Gronwald et al., 2018), particularly depression (Budde et al., 2025). It is estimated that the economic ramifications of physical inactivity exceed $500 billion annually in the United States, encompassing medical care, productivity loss, and workers’ compensation (Duijvestijn et al., 2023; Chenoweth and Leutzinger, 2006). However, understanding how to maintain long-term physical activity, especially among initially inactive individuals, remains elusive.

For many, depression plays a pivotal role in the reduction and lack of sustained physical activity. Depression is highly prevalent (Brody et al., 2018) and not only enhances the risk of cancers, heart diseases, diabetes, and chronic pains (Srinivasan et al., 2025; Saleh et al., 2024; Rafiei et al., 2023; Cooper et al., 2023; Habib et al., 2022; Roughan et al., 2021) but also correlates with decreased physical activity (Roshanaei-Moghaddam et al., 2009; Okoro et al., 2014). Studies spanning diverse groups including elderly individuals and patients with cancer or diabetes have identified depressive symptoms as a predictor of lower adherence to exercise programs (Sumlin et al., 2014; Chipperfield et al., 2013; Jefferis et al., 2014). A core feature of depression is a dysregulated affect (American Psychiatric Association, 2022), characterized by lower levels of positive emotions, which are known to be associated with reduced physical activity (Jekauc and Brand, 2017; Pasco et al., 2011). While exercise generally leads to positive emotions after completion (Reed and Ekkekakis, 2015), the affective experience varies during the activity (Ekkekakis, 2017; Ekkekakis et al., 2020). Specifically, exercise is associated with physiological sensations (e.g., shortness of breath, sweating, increased heart rate, muscle pain) that can be perceived as unpleasant. The ability to tolerate these internal bodily sensations and redirect attention away from them plays a key role in making exercise feel more enjoyable (Ekkekakis, 2003). However, individuals with depression exhibit a well-documented negative attentional bias, characterized by an increased tendency to selectively attend to and amplify negative stimuli (Mennen et al., 2019). This cognitive pattern likely extends to interoceptive sensations during exercise, potentially heightening the perception of discomfort and contributing to a more aversive experience of exercise. Indeed, physical activity enjoyment has been shown to be lower in individuals with depression (Abrantes et al., 2017). Since the affective experience of exercise is a key predictor of long-term adherence (Stevens et al., 2020; Rhodes and Kates, 2015), innovative strategies for improving the affective experience of exercise in individuals with depression may be essential for sustained physical activity in this population.

Neurophysiological mechanisms of the affective responses to exercise point to potential intervention strategies. Studies using electroencephalography (EEG) have shown that lateralization of brain activity predicts the affective experiences of exercise, i.e., greater activation in the left prefrontal cortex relative to the right is associated with more positive affective responses to aerobic exercise (Hall et al., 2010; Hall et al., 2007; Woo et al., 2009; Silveira et al., 2019). This pattern is particularly relevant for individuals with depression, who exhibit reduced activation in the left dorsolateral prefrontal cortex (DLPFC) and increased activity in the right DLPFC, a neural asymmetry associated with difficulties in affect regulation (Grimm et al., 2008; Herrington et al., 2010). Given that a positive affective experience during exercise is a key predictor of long-term adherence, interventions that enhance left DLPFC activation may help mitigate the negative emotional response to exercise in individuals with depression, thereby promoting greater physical activity engagement. One promising approach for targeting this neural imbalance is transcranial direct current stimulation (tDCS), a safe, noninvasive brain stimulation technique that modulates neuronal excitability through low-intensity electrical current applied to the scalp (Brunoni et al., 2011). A substantial body of evidence supports the efficacy of tDCS in enhancing cognitive and affective processes across various populations (Narmashiri and Akbari, 2023; Zheng et al., 2024). Specifically, tDCS targeting the left DLPFC has demonstrated potential in improving cognitive control over negative emotions, particularly in individuals with depression (Wiegand et al., 2019; Feeser et al., 2014; Plewnia et al., 2015; Wolkenstein and Plewnia, 2013). Given its ability to strengthen cognitive control over affective experiences, tDCS may be a valuable intervention for improving the affective response to exercise, thereby increasing the likelihood of sustained physical activity participation in this population.

The application of tDCS in the realm of exercise science has gained attention in the past decade, with numerous studies illustrating its potential in augmenting physical training outcomes (Angius et al., 2015; Hendy and Kidgell, 2014; Kaski et al., 2014; Etemadi et al., 2023; Machado and Amiri, 2023; Isis et al., 2023), including improvements in muscle strength (Lattari et al., 2018). Predominantly focusing on the motor cortex, tDCS has been observed to enhance mobility and physical functionality in conditions such as cerebral palsy and stroke (Duarte Nde et al., 2014; Park et al., 2015). Meanwhile, tDCS targeting the DLPFC has been associated with varying outcomes, including increased oxygen consumption post-exercise, reduced feelings of hunger post-activity, improved gait in individuals with Parkinson’s disease (Etemadi et al., 2023; Montenegro et al., 2014; Montenegro et al., 2012; Wong et al., 2024) as well as improved cognitive functioning (Thomas et al., 2021). Yet, aside from a few exceptions, there is a lack of studies investigating the impact of tDCS on the emotional experience of exercise or on long-term exercise commitment in individuals prone to negative affect like those with depressive symptoms. Considering the pivotal role of the affective experience in determining long-term physical activity, we theorized that integrating tDCS (targeting the left DLPFC) with an exercise regimen might offer a promising avenue to bolster exercise adherence.

The purpose of the current pilot study was to determine the feasibility of tDCS (anode over left DLPFC) delivered in combination with an 8-week aerobic exercise intervention for increasing aerobic exercise (AE) in individuals with elevated depressive symptoms. Feasibility is determined via session attendance, assessment retention, blinding integrity, and device-wear compliance. We also examine within‐subject effect size estimates (i.e., Cohen’s d) for changes in objective and self-reported physical activity, aerobic capacity, and mood from pre‐ to post‐intervention and at 6‐month follow-up for each treatment group. In doing so, we may be able to obtain a preliminary determination of whether combining tDCS with exercise enhances physical activity and affective outcomes relative to exercise alone. These insights will directly inform the design and sample size planning of a future definitive trial.

## 2 Materials and methods

### 2.1 Study design

The present study was a two-arm parallel pilot randomized controlled trial (RCT). The protocol paper has previously published (Abrantes et al., 2022). The Butler Hospital Institutional Review Board approved all study procedures and this RCT was registered on clinicaltrials.gov [NCT03178903]. The first participant was enrolled in June 2017 and the final participant completed the last assessment in October 2019. CONSORT guidelines for pilot randomized trials was followed for reporting the methods and results of this study (Eldridge et al., 2016).

### 2.2 Participants

Inclusion criteria were: (1) age 18–65, (2) low engagement in physical activity (less than 90 min of moderate-intensity exercise/week for the past 6 months), (3) elevated depressive symptoms (Center for Epidemiological Studies Depression Scale [CES-D] score at least 10) (Radloff, 1977), (4) interest in beginning an exercise program in the next month, and (5) able to walk one mile on a treadmill.

Exclusion criteria were: (1) history of mania, hypomania, or psychotic disorder; (2) current diagnosis of anorexia nervosa, bulimia nervosa, or other eating disorder for which an exercise intervention would be contraindicated; (3) moderate or severe substance use disorder; (4) suicidality or homicidality; (5) untreated major depressive disorder; (6) physical disabilities or medical problems that precluded participation in moderate intensity exercise (i.e., physician denied medical clearance), were contraindicated with tDCS (e.g., seizure disorder), or that might otherwise have interfered with study procedures (e.g., contagious skin disease); (7) pregnancy or breastfeeding at the time of enrollment, or intent to become pregnant during the subsequent 8 weeks; (8) presence of a pacemaker or metal implanted within the cranial cavity; and (9) psychiatric medication changes within 6 weeks prior to study entry.

### 2.3 Intervention procedures and descriptions

Both interventions included supervised AE with half the participants receiving tDCS prior to each AE session (AE + tDCS) and half receiving sham prior to their AE sessions (AE + sham).

#### 2.3.1 tDCS or sham

Participants received 20 min of either active or sham tDCS at an intensity of 1 mA for active tDCS. Each carbon rubber anode and cathode electrode was inserted in a 5 × 5 cm (25 cm^2^) rectangular saline-soaked sponge (0.4 A/m2 current density). The anode was placed over left DLPFC (F3 on the EEG 10–20 system) and the reference electrode (cathode) over the contralateral (right) supraorbital region. Stimulation was delivered by a battery-driven, constant current stimulator (NeuroConn DC Stimulator Plus) which included a blinded study option such that participant-specific codes were entered into the device to deliver active or sham stimulation without unblinding the administrator. Sham stimulation matched the active stimulation in timing, and used a “ramp up/ramp down” approach, with delivery of current starting (gradually ramped up) and then ending (gradually ramped down) over 30 s, and 15 milliseconds 10 μA current pulse applied every 550 milliseconds, with a 3-millisecond peak current to ensure adequate impedance. Participants received the AE session immediately following the active or sham stimulation session. Participants attend stimulation plus AE sessions 3 days per week for 8 weeks.

#### 2.3.2 AE

All participants received supervised AE sessions three times/week at Butler Hospital’s Fitness Facility. Sessions began with a 5-min warm-up followed by 20–30 min of moderate-intensity AE on a treadmill and concluded with a 5-min cool-down. Exercise physiologists provided supervision and monitored participants’ heart rates to ensure they remained within the moderate intensity range (64%–76% of age predicted maximal heart rate), adhering to recommendations for standardization of exercise intensity for effective outcomes (Gronwald et al., 2019). Participants were encouraged to gradually increase the amount of physical activity they completed outside of supervised sessions to reach the recommended 150 total weekly minutes of moderate-intensity AE (Piercy et al., 2018). Resources were provided to facilitate exercise outside the study sessions (e.g., exercise videos), including strategies for integrating PA into their daily lives.

### 2.4 Measures

#### 2.4.1 Physical activity and fitness

Moderate-to-vigorous physical activity (MVPA) minutes/week were measured objectively with accelerometry at baseline and the 3-month follow-up. At each of these timepoints, participants were instructed to wear an Actigraph wGT3X-BT device on their hip for 7 days. Wear time of 8 hours or more was considered a valid day (Choi et al., 2011) and MVPA minutes/week were calculated for participants with three or more valid days. The Short Form of the International Physical Activity Questionnaire (IPAQ) was used to capture self-reported physical activity and sedentary time (Lee et al., 2011). The Rockport 1-mile walk test, during which participants walk one mile on a treadmill as quickly as possible, was used to measure cardiorespiratory fitness (Pober et al., 2002).

To capture physical activity levels outside the intervention sessions, participants in each condition were asked to wear a Fitbit Alta during waking hours for the entirety of the 8-week intervention period. Average steps/day for each week of the intervention were collected for days when the participant wore the Fitbit for at least 8 h.

#### 2.4.2 Physical activity motivation and enjoyment

The Behavioral Regulation in Exercise (BREQ-2), a 19-item self-report scale, was used to measure physical activity motivation (Markland and Tobin, 2016). The Physical Activity Enjoyment Scale (PACES), an 18-item self-report scale, was used to measure level of physical enjoyment (Kendzierski and DeCarlo, 1991).

#### 2.4.3 Depression and affect

The CES-D, a 20-item self-report scale, was used to assess severity of depressive symptoms (Radloff, 1977). This measure was used both for inclusion in the study (cutoff: 10) as well as to track changes in depressive symptoms. The Positive and Negative Affect Schedule (PANAS), a 20-item measure, was used to assess positive and negative affect (Watson et al., 1988).

### 2.5 Procedure

Potential participants were recruited through social media advertisements and brochures with taglines about “a study to help people stick with exercise” that were posted hospital and community locations. Interested individuals were screened via phone to assess current level of physical activity and depressive symptoms. Potentially eligible participants from the phone screen were subsequently scheduled for a comprehensive baseline assessment to confirm eligibility. After obtaining informed consent, study staff administered select modules from the Structured Clinical Interview for DSM-5 (American Psychiatric Association, 2022) to assess the presence/absence of an MDD diagnosis and any of the exclusory diagnoses. A release of information was obtained from participants to contact primary care providers for medical clearance. At the completion of the baseline assessment, participants were randomized 1:1–8 weeks of AE + tDCS or AE + sham stratified by MDD diagnosis. All participants, assessors, and tDCS interventionists were blind to group assignment. Assessments were conducted at baseline, end of treatment (8 weeks), 3-month follow-up (12 weeks), and 6-month follow-up (24 weeks). Because PA accelerometry was only collected at EOT and 6-month follow-up, we only report outcomes at these timepoints. Participants were paid $50 for each completed assessment.

### 2.6 Data analysis

All analyses followed an intention-to-treat framework, with key feasibility metrics (session attendance, assessment completion, blinding integrity, device wear compliance, and valid accelerometry days) summarized descriptively. Within-subject changes from baseline to end of treatment (EOT; 8 weeks) and baseline to 6-month follow-up (i.e., 4 months after the end of the intervention) were quantified by Cohen’s d effect sizes (and 95% confidence intervals) on physical activity variables as well as mood and affect outcomes (i.e., depression, physical activity enjoyment, positive affect and negative affect), with pairwise exclusion of missing data. For accelerometry outcomes, we used the average daily steps and average daily MVPA minutes across all available days at each time point. For the IPAQ, we used the average weekly MVPA minutes across all available time points. For the 1-mile walk test, we used the peak VO2 from the assessment closest to each time point. Effect sizes were interpreted using conventional benchmarks (d ≈ 0.2 = small, d ≈ 0.5 = medium, d ≈ 0.8 = large) (Cohen, 1988), providing a standardized gauge of change magnitude without formal hypothesis testing. All analyses were conducted using SPSS Version 31.

## 3 Results

### 3.1 Participant characteristics

**FIGURE 1:**
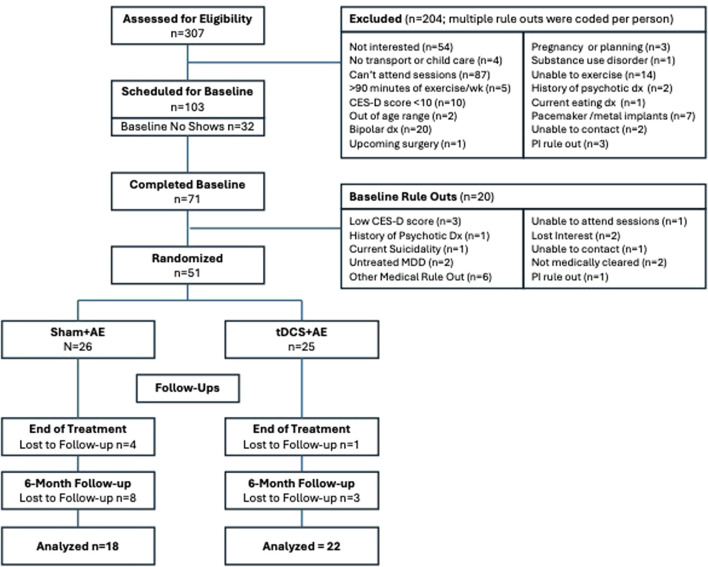
RCT Consort diagram.

Fifty-one individuals were randomized to AE + tDCS (n = 25) or AE + sham (n = 26). See the Consort Diagram (Figure 1) for a description of the study recruitment. There were no significant differences between the AE + tDCS and AE + sham groups on demographic variables or baseline levels of physical activity, cardiorespiratory fitness, mood, or affect (all *p*s > 0.05; see Table 1). The sample was predominantly White (90.2%) and female (86.3%) with a mean age of 49.5 years (SD = 10.4). The average BMI was 35.1 (SD = 8.0), indicating that the sample was, on average, obese. At baseline, participants reported an average of 21.8 min of moderate-to-vigorous physical activity (MVPA) per week on the IPAQ and wore the accelerometer for an average of 6.1 days (SD = 1.5). Using accelerometry, the average daily step count at baseline was 4,493 (SD = 1,844) and the average daily MVPA was 17.3 min (SD = 13.3). The average peak VO2 was 18.9 (SD = 7.8). The average CES-D score was 22.3 (SD = 10.7), indicating elevated levels of depressive symptoms.

**TABLE 1:** Baseline characteristics by intervention group.

	Sample (n = 51)	Intervention group	
		AE + tDCS (n = 25)	AE + Sham (n = 26)
Demographics			
Age (Years)	49.5 (±10.4)	48.9 (±9.7)	50.1 (±11.1)
Gender (Female)	44 (86.3%)	21 (80.8%)	23 (92.0%)
Race			
White	46 (90.2%)	23 (88.5%)	23 (92.0%)
Black or African American	3 (5.9%)	2 (8.0%)	1 (3.8%)
Other	2 (3.9%)	0 (0%)	2 (7.7%)
Ethnicity (Latinx)	4 (8.0%)	0 (0%)	4 (15.4%)
Body Mass Index (BMI)	35.1 (±8.0)	35.4 (±6.7)	34.9 (±9.2)
Physical Activity			
Accelerometry steps/day	4,493 (±1844)	4,745 (±2011)	4,230 (±1,656)
Accelerometry MVPA minutes/day	17.3 (±13.3)	15.0 (±11.0)	19.7 (±15.3)
Cardiorespiratory Fitness (peak VO2)	18.9 (±7.8)	18.2 (±7.8)	19.5 (±7.9)
Physical Activity Questionnaire – IPAQ)	21.8 (±69.5)	11.6 (±21.4)	31.5 (±94.9)
Mood and Affect Measures			
Depression Scores (CESD)	22.3 (±10.7)	22.9 (±10.0)	21.8 (±11.6)
Physical Activity Enjoyment (PACES)	65.0 (±17.7)	60.5 (±17.1)	69.4 (±17.4)
PANAS Positive	26.0 (±8.6)	25.5 (±9.3)	26.6 (±9.3)
PANAS Negative	16.3 (±5.5)	15.8 (±5.4)	16.8 (±5.6)

### 3.2 Feasibility metrics: adherence and compliance with study procedures

Participants attended a mean of 15.5 (SD = 6.4) sessions during the 8-week intervention (from a total possible 24 sessions; 65%). There were no differences in session attendance between groups. Follow-up rates were good (90.2% completed end-of-treatment (EOT) and 78.4% completed 6-month follow-up) and blinding was successful as belief about receiving active stimulation was comparable across conditions (41.6% of the active tDCS group and 36.4% of the sham group).

There was high level of Fitbit wear during the intervention, with 84.3% of the sample wearing it the entire 8 weeks of the intervention (76.9% of AE + sham and 92% of AE + tDCS; chisquare = 2.19; p = 0.13). There were no group differences in the percent who provided at least 3 days of valid accelerometry (GT3X) at EOT (70%) and 6-month follow-up (51%).

### 3.3 Physical activity and affective outcomes

End of Treatment (See Table 2). In the active tDCS arm, within-subject effect sizes were small to medium for accelerometer-derived daily steps (d = 0.36) and MVPA (d = 0.34). Cardiorespiratory fitness exhibited a large improvement (peak VO_2_, d = 0.99), while self-reported MVPA increased with a moderate‐to‐large effect (d = 0.78). Depressive symptoms declined with a large effect (CES-D, d = −1.00), and exercise enjoyment increased moderately (PACES, d = 0.62). Positive affect showed a small effect (PANAS-Positive, d = 0.22), and negative affect was essentially unchanged (PANAS-Negative, d = −0.14).

**TABLE 2:** End-of-Treatment within group changes in outcomes (paired sample t-tests).

Outcome	Baseline (SD)	EOT (SD)	Cohen’s D (95% confidence Interval)
Accelerometry steps/day			
AE + tDCS (n = 19)	4,826 (1981)	5,251 (1747)	0.36 (−0.11 to 0.83)
AE + Sham (n = 14)	4,387 (1727)	3,993 (1,498)	−0.28 (−0.81 to 0.26)
Accelerometry MVPA minutes/day			
AE + tDCS (n = 19)	14.9 (9.2)	18.4 (9.9)	0.34 (−0.13 to 0.80)
AE + Sham (n = 14)	22.5 (17.2)	22.8 (20.5)	0.02 (−0.48 to 0.53)
Cardiorespiratory Fitness (peak VO2)			
AE + tDCS (n = 20)	18.0 (7.4)	21.8 (6.1)	0.99 (0.44–1.52)
AE + Sham (n = 17)	18.8 (7.8)	22.8 (7.9)	1.18 (0.54–1.79)
Self-reported MVPA/week (International Physical Activity Questionnaire – IPAQ)			
AE + tDCS (n = 21)	11.5 (21.8)	110.8 (124.2)	0.78 (0.32–1.23)
AE + Sham (n = 21)	10.5 (23.5)	75.2 (78.0)	0.90 (0.38–1.40)
Depression Scores (CES-D)			
AE + tDCS (n = 24)	23.4 (9.9)	13.8 (10.6)	−1.0 (−1.5 to −0.53)
AE + Sham (n = 22)	20.7 (11.8)	12.1 (5.7)	−0.88 (−1.37 to −0.38)
Physical Activity Enjoyment (PACES)			
AE + tDCS (n = 24)	61.5 (16.7)	76.9 (21.1)	0.62 (0.17–1.05)
AE + Sham (n = 22)	69 (17.9)	80 (19.2)	0.49 (0.04–0.93)
PANAS Positive			
AE + tDCS (n = 24)	25.2 (7.9)	27.3 (10.2)	0.22 (−0.18 to 0.63)
AE + Sham (n = 22)	26.7 (9.9)	26.8 (9.9)	0.02 (−0.40 to 0.43)
PANAS Negative			
AE + tDCS (n = 24)	15.8 (5.5)	15.0 (7.6)	−0.14 (−0.54 to 0.26)
AE + Sham (n = 22)	16.2 (5.5)	13.1 (3.0)	−0.53 (−0.97 to −0.07)

In the sham arm, cardiorespiratory fitness also improved markedly (peak VO_2_, d = 1.18), and self-reported MVPA rose with a moderate‐to‐large effect (d = 0.90). Depressive symptoms decreased with a large effect (d = −0.88), and enjoyment increased moderately (d = 0.49). Effects on accelerometer-derived daily steps (d = −0.28) and MVPA (d = 0.02) as well as positive affect (d = 0.02) were negligible, while negative affect showed a moderate reduction (d = −0.53).

Six-Month Follow-up (See Table 3). At 6 months, in the context of much smaller sample with objective data, the active tDCS group maintained small‐to‐medium improvements in activity and fitness: accelerometer-derived steps/day (d = 0.35) and MVPA (d = 0.42), as well as peak VO_2_ (d = 0.79). Self-reported MVPA remained elevated (d = 0.69), depressive symptoms continued to show a reduction (d = −0.74), and enjoyment remained improved (d = 0.70). Affect remained largely stable (PANAS-Positive d = 0.27; PANAS-Negative d = −0.13).

**TABLE 3:** 6-Month within group changes in outcomes (paired sample t-tests).

Outcome	Baseline (SD)	6-Month (SD)	Cohen’s D (95% confidence Intervals)
Accelerometry steps/day			
E + tDCS (n = 13)	4,300 (1832)	4,916 (1869)	0.35 (−0.22 to 0.91)
AE + Sham (n = 11)	4,875 (1761)	4,805 (1954)	−0.05 (−0.64 to 0.54)
Accelerometry MVPA minutes/day			
AE + tDCS (n = 13)	12.6 (7.7)	17.9 (11.0)	0.42 (−0.15 to 0.98)
AE + Sham (n = 11)	24.6 (19.1)	29.5 (29.3)	0.33 (−0.29 to 0.93)
Cardiorespiratory Fitness (peak VO2)			
AE + tDCS (n = 10)	19.1 (4.9)	21.7 (4.4)	0.79 (0.06–1.49)
AE + Sham (n = 9)	19.1 (7.4)	22.1 (5.6)	1.1 (0.21–1.88)
Self-reported MVPA/week (International Physical Activity Questionnaire – IPAQ)			
AE + tDCS (n = 22)	12.5 (22.6)	107.58 (132.9)	0.69 (0.22–1.15)
AE + Sham (n = 17)	10.5 (23.5)	57.4 (60.1)	0.80 (0.24–1.34)
Depression Scores (CESD)			
AE + tDCS (n = 22)	22.5 (9.9)	15.1 (11.7)	−0.74 (−1.21 to −0.26)
AE + Sham (n = 18)	20.7 (11.8)	11.9 (8.0)	−0.59 (−1.08 to −0.08)
Physical Activity Enjoyment (PACES)			
AE + tDCS (n = 22)	61.9 (17.0)	77.6 (19.9)	0.70 (0.22–1.16)
AE + Sham (n = 18)	68.5 (18.9)	74.6 (23.0)	0.26 (−0.22 to 0.72)
PANAS Positive			
AE + tDCS (n = 22)	26.1 (7.6)	28.4 (10.2)	0.27 (−0.16 to 0.61)
AE + Sham (n = 18)	27.6 (10.3)	28.4 (11.0)	0.10 (−0.37 to 0.56)
PANAS Negative			
AE + tDCS (n = 22)	15.0 (5.0)	14.5 (5.4)	−0.13 (−0.55 to 0.29)
AE + Sham (n = 18)	15.3 (4.6)	13.1 (2.6)	−0.41 (−0.88 to 0.08)

Sham participants, also a smaller sample size with objective data at 6-months, demonstrated a large fitness gain (peak VO_2_, d = 1.10) and moderate increases in self-reported MVPA (d = 0.80), but minimal change in daily steps (d = −0.05). Depressive symptoms and enjoyment showed small‐to-medium improvements (d = −0.59 and d = 0.26, respectively), with negligible effects on affect (PANAS-Positive d = 0.10; PANAS-Negative d = −0.41).

**FIGURE 2:**
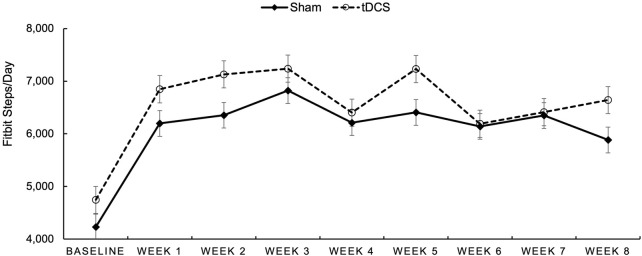
Average Fitbit-measured steps/day for participants randomized to Sham (dotted line with open circles) and to tDCS (solid line with diamonds) over each week of the intervention period.

During Intervention Steps per Day (See Figure 2). We examined group differences in average steps/day collected with Fitbits for each week of the intervention. In Figure 2, baseline steps per day were derived from the 7-day accelerometry wear time at baseline and are depicted in the figure to provide this reference. However, the remaining steps/day for the entire 8 weeks of the intervention are graphed utilizing data from the daily wear of the Fitbit device. Participants appeared to average between 6,000 and 7,000 steps per day (Fitbit derived) during the intervention period with participants in the AE + tDCS group demonstrating consistently higher means relative to the sham group.

## 4 Discussion

This pilot randomized controlled trial examined the feasibility of combining tDCS with aerobic exercise (AE) for increasing physical activity in adults with elevated depressive symptoms. Participants were randomized to receive either active or sham tDCS targeting left dorsolateral prefrontal cortex (DLPFC) prior to supervised AE sessions three times per week for 8 weeks. The results indicate that the intervention was feasible and we observed within group small-to-medium effects for increased accelerometry-derived changes in steps/day and MVPA at EOT in the active tDCS group but not sham. Moderate to large increases in fitness, self-reported MVPA, and physical activity enjoyment as well as decreases in depression were observed within each group.

The intervention’s adherence rates and participant compliance were moderate and comparable between the AE + tDCS and AE + sham groups in session attendance. Follow-up rates were excellent at EOT and acceptable at 6-months. We observed good compliance with Fitbit use during the intervention but lower rates of compliance with the 7-day wear of the GT3X accelerometer. Integrity of the blind was upheld as groups were comparable on the extent to which they believed they received active stimulation, thereby ensuring observed effects are likely attributable to active tDCS rather than participant expectations (Budde et al., 2023). Overall, these findings are encouraging for future studies as it demonstrates the feasibility of incorporating tDCS into standard exercise programs without compromising participant engagement. In addition, self-initiated or home-based tDCS has seen significant development, particularly in individuals with depression (Cappon et al., 2021). With proper training and oversight, patients could potentially effectively administer tDCS at home.

The current findings are consistent with previous research demonstrating the potential of tDCS to augment the effects of exercise on physical function and mobility (Etemadi et al., 2023; Wong et al., 2024; Thomas et al., 2021). However, the present study extends this literature by examining the effects of tDCS on objectively measured physical activity in individuals with elevated depressive symptoms, a population that is at increased risk for physical inactivity (Roshanaei-Moghaddam et al., 2009; Okoro et al., 2014; Goodwin, 2003). Both groups showed moderate to large effects for decreases in depression and large effects for increases in fitness. The active tDCS group showed small to medium effect sizes for increases in MVPA at both EOT and 6-month and the sham group showed moderate effects for increases in MVPA at 6-month. The fact that both groups showed similar changes in these outcomes is not too surprising given that both conditions (the active and sham tDCS) engaged in the same supervised program of moderate-intensity aerobic exercise. That is, participation in the AE alone is expected to produce these changes.

However, the observed effect of tDCS (but not sham) on step count (i.e., small to moderate effect size), is particularly noteworthy and suggests that increases in steps were likely due to everyday physical activity outside the supervised weekly sessions. The distinction between structured exercise and general physical activity, such as daily step counts, is an important one for understanding the broader impact of interventions (Budde et al., 2016). That is, participants may have found ways to incorporate more steps into their everyday routine; this is consistent with an approach called “lifestyle physical activity” (Farris and Abrantes, 2020) which emphasizes increasing activity throughout the entire day, and not just in the context of isolated exercise bouts. This finding was unexpected but points to a promising result, given the comparable mental and physical health benefits of lifestyle physical activity (Farris and Abrantes, 2020; Ahmadi et al., 2023; Dunn et al., 1999; Abrantes et al., 2024). Indeed, even though observed increases in steps/day were modest (∼400 at EOT and ∼600 at 6-months), these increases align with levels identified in the literature for the minimally clinically important difference in various health outcomes (Demeyer et al., 2016; Polgar et al., 2021), including reducing risk of all-cause morbidity and mortality (Stens et al., 2023). Therefore, the broader implications for daily activity patterns, potentially influenced by underlying biological rhythms and cognitive function (Yalcin et al., 2022) warrant further exploration.

Of note, effect sizes were much larger, in each group, for self-reported MVPA relative to objectively measured physical activity via accelerometry. This disparity is a common finding in physical activity research and may be attributed to several factors. Participants might overestimate their activity levels due to social desirability bias, or they may find it challenging to accurately recall the duration and intensity of their physical activity over a given period (Dowd et al., 2018). Objective measures, while less susceptible to recall bias, can also have limitations, such as issues with wear time compliance or the inability to capture all forms of activity (Migueles et al., 2017). Our observation of higher Fitbit-derived daily step counts (6,000–7,000 steps per day) compared to the accelerometer-derived (∼4,500 steps per day) further illustrates this variability across objective measurement devices and contexts. Future research should consider employing a combination of comprehensive objective measures and validated self-report tools, along with strategies to maximize wear compliance for objective devices, to gain a more complete understanding of physical activity changes.

The comparable changes in self-reported depressive symptoms, positive affect, or negative affect for each group suggests that while tDCS may not influence overall mood above and beyond the effects experienced with aerobic exercise. Therefore, it is likely that the effects of tDCS on increased step counts may be mediated by factors that extend beyond mood, such as cognitive control or motivation (Wiegand et al., 2019; Feeser et al., 2014; Plewnia et al., 2015; Wolkenstein and Plewnia, 2013). Indeed, the mechanisms by which tDCS may enhance the effects of exercise on physical activity are not fully understood and previous research highlights the complexity of physiological responses to exercise in individuals with depression (Lamego et al., 2015). As DLPFC plays a role in carrying out a range of cognitive functions, more work is needed to clarify the specific neurocognitive mechanism by which DLPFC-targeted stimulation may act to enhance engagement in physical activity. Further, evidence suggests tDCS induces cortical neuroplasticity, leading to long-term changes in brain structure and function (Jog et al., 2023). When combined with a physical activity intervention, neuroplastic mechanisms may enhance the network changes needed to support sustained physical activity (Steinberg et al., 2018). However, these proposed mechanisms, while grounded in a strong scientific premise, are still speculative and require rigorous testing that incorporates neuroimaging modalities to confirm neural target engagement of tDCS.

Despite the promising findings, several limitations should be noted. First, as the primary aim of this pilot study was to assess feasibility of tDCS + AE, the sample size was not fully powered to detect significant group differences. The sample was also predominantly White and female, which limits the generalizability of the findings to other populations. Additionally, although adherence to the Fitbit was high during the intervention period, there were missing accelerometry and fitness data at the end-of-treatment and follow-up assessments, which may have introduced bias in interpreting the results. Future research should address these limitations by including larger and more diverse samples, as well as strategic efforts to increase compliance with objective measurements of physical activity and fitness.

In conclusion, this pilot RCT provides preliminary evidence for the feasibility of combining tDCS with AE for increasing physical activity in adults with elevated depressive symptoms. The findings suggest that tDCS may be a promising adjunct to moderate-intensity supervised exercise interventions for promoting lifestyle physical activity in this population. Future research is needed to replicate and extend these findings, as well as to elucidate the mechanisms by which tDCS enhances the effects of exercise on physical activity.

## Data Availability

The raw data supporting the conclusions of this article will be made available by the authors, without undue reservation.
